# Holo-lactoferrin: the link between ferroptosis and radiotherapy in triple-negative breast cancer

**DOI:** 10.7150/thno.52028

**Published:** 2021-01-01

**Authors:** Zheng Zhang, Menglan Lu, Cailong Chen, Xing Tong, Yunhong Li, Kai Yang, Haitao Lv, Jiaying Xu, Liqiang Qin

**Affiliations:** 1 Department of Nutrition and Food Hygiene, School of Public Health, Soochow University, 199 Renai Road, Suzhou 215123, China; 2 Center of Child Health Management, Children's Hospital of Soochow University, Suzhou, 215025, China; 3 Laboratory Center, Medical College of Soochow University, Suzhou, 215123, China; 4 State Key Laboratory of Radiation Medicine and Protection, Collaborative Innovation Center of Radiation Medicine of Jiangsu Higher Education Institutions, School of Radiation Medicine and Protection, Soochow University, 199 Renai Road, Suzhou 215123, China

**Keywords:** Holo-Lf, ferroptosis, radiotherapy, triple-negative breast cancer, hypoxia

## Abstract

**Rationale:** Iron-saturated Lf (Holo-Lactoferrin, Holo-Lf) exhibits a superior anticancer property than low iron-saturated Lf (Apo-Lf). Ferroptosis is an iron-dependent cell death characterized by the accumulation of lipid peroxidation products and lethal reactive oxygen species (ROS). Radiotherapy also exerts its therapeutic effect through ROS.

**Methods:** The effect of different iron-saturated Lf on ferroptosis and radiotherapy were tested on triple-negative breast cancer (TNBC) cell line MDA-MB-231 and non-TNBC cell line MCF-7.

**Results:** Holo-Lf significantly increased the total iron content, promoted ROS generation, increased lipid peroxidation end product, malondialdehyde (MDA), and enhanced ferroptosis of MDA-MB-231 cells. By contrast, Apo-Lf upregulated SLC7a11 expression, increased GSH generation and inhibited ferroptosis of MDA-MB-231 cells. However, non-TNBC MCF-7 cells were resistant to Holo-Lf-induced ferroptosis because MCF-7 cells have a higher redox balance capacity than MDA-MB-231 cells. More importantly, Holo-Lf downregulated HIF-1α expression, ameliorated the hypoxia microenvironment in subcutaneous MDA-MB-231 tumors, and promoted radiation-induced DNA damage to hypoxic MDA-MB-231 cells. Finally, the efficacy of radiotherapy to MDA-MB-231 tumors was enhanced by Holo-Lf.

**Conclusion:** Holo-Lf could induce ferroptosis in MDA-MB-231 cells and sensitize MDA-MB-231 tumors to radiotherapy.

## Introduction

Triple-negative breast cancer (TNBC) is a subtype of breast cancer in which estrogen receptor (ER), progesterone receptor (PR), and human epidermal growth factor receptor type 2 (HER 2) are not expressed. Thus, TNBC patients derive no benefit from endocrine therapy or trastuzumab. Moreover, the breast cancer susceptibility gene 1 (BRCA1) mutations are present at high frequency in TNBC patients, resulting in increased aggressiveness of TNBC 1. All these features contribute to the poor prognosis of TNBC patients 2.

Lactoferrin (Lf) is a pleiotropic protein that functions in multiple physiological processes and is beneficial in many diseases. It is implicated in infectious diseases 3-6, obesity 7, type 2 diabetes 8, and cardiovascular diseases 9, and its potential applications in cancer therapy have attracted much attention 10, 11. In a randomized control trial, oral human recombinant Lf significantly increased overall survival by 65% compared with the placebo in patients with advanced non-small-cell lung carcinoma 12. Furthermore, cellular uptake of cancer theranostic agents (quantum dots and celecoxib/honokiol nanoparticles/doxorubicin) was elevated upon conjugation with Lf, and their therapeutic effect on breast cancer was also enhanced 13, 14.

The extent of iron-saturation influences anticancer features of Lf. Iron-saturated Lf (Holo-Lf), but not low iron-saturated Lf (Apo-Lf), inhibits the proliferation of Caco-2 cells 15. A study in mice suggested that the Holo-Lf diet prior to chemotherapy (paclitaxel/doxorubicin) eradicated the EL-4 tumor and Lewis lung carcinoma, whereas the Apo-Lf diet did not augment the chemotherapy effect 16. In another study, when MDA-MB-231 and MCF-7 cells were incubated with Apo-Lf and Holo-Lf, a higher percentage of cell death was induced by Holo-Lf than Apo-Lf in both cell lines 17. Thus, Holo-Lf has a superior anticancer property over Apo-Lf, but the mechanism for the differential anticancer potency of different iron-saturated forms of Lf remains undefined.

Radiotherapy exerts its therapeutic effect mainly through ionizing radiation, producing reactive oxygen species (ROS) and free radicals to break chemical bonds and initiate a series of events that result in DNA damage. Oxygen can react with free radicals to yield a stable change in the chemical composition of DNA damage. However, the hypoxia microenvironment in solid malignancies can influence the fixation of DNA damage and result in radiotherapy resistance of tumors 18. Moreover, the hypoxia microenvironment stabilizes the hypoxia-inducible factor (HIF), induces the hypoxia-related metastasis of cancer, and ablates the treatment effect of radiotherapy.

Ferroptosis is iron-dependent cell death characterized by the accumulation of ROS and lipid peroxidation products 19. Ferroptosis has been shown to enhance the cytotoxicity of chemotherapy drugs (cisplatin and doxorubicin) to chemotherapy-resistant cancer cells 20-23, sensitize cancers to radiotherapy 24, and amplify the efficacy of photothermal and photodynamic therapy to cancers 25-30. Therefore, targeting the ferroptosis pathway has been proposed as a new strategy to enhance therapeutic modalities for cancer 31-34. More importantly, the FDA-approved superparamagnetic iron oxide nanoparticles drug (ferumoxytol) has been reported to trigger ferroptosis for killing cancer cells 35. It remains unclear whether Holo-Lf could induce ferroptosis and improve the radiosensitivity of TNBC still. The present study aimed to investigate the potential differential effects of different iron-saturated forms of Lf on ferroptosis and radiotherapy in TNBC.

## Materials and Methods

### Cell culture

Breast cancer cell lines, MDA-MB-231 and MCF-7 cells, were purchased from ATCC. The cells were cultured in DMEM high glucose medium (Hyclone) supplemented with 10% (V/V) FBS and 1% penicillin and streptomycin at 37 °C in a humidified 5% CO_2_ atmosphere. For confocal microscopy, the cells were seeded in the confocal dish (2×10^4^ cells each dish) for 24 h and then incubated with different interventions. For flow cytometry, the cells were seeded in 6-well plates at a density of 2×10^5^ cells/well. The hypoxic condition was achieved by incubating cells in the hypoxia chamber with 94% N_2_, 5% CO_2_, and 1% O_2_. The high hydrogen peroxide level after hypoxia culture was simulated by incubating cells with 200 µM H_2_O_2_ for 1 h. Cell viability was measured by the cell counting (CCK-8) (MedChemExpress) assay. For the CCK-8 assay, cells were seeded in 96-well plates at a density of 8×10^3^ cells/well and then incubated with different treatments. After the addition of 10 µL CCK-8 assay buffer per well, the absorbance at 450 nm was measured.

### Breast cancer model

MDA-MB-231 cells were resuspended in pre-cooled Matrigel (BD Bioscience, USA). Female BALB/C nude mice (3-5 weeks) were subcutaneously injected with 50 µL of MDA-MB-231 cells (2×10^6^) in the back. All animal experiments were performed in accordance with the Experimental Animal Administrative Committee of Soochow University guidelines.

### Preparation of Apo-Lf

Apo-Lf was prepared as previously described with modifications 36, 37. Briefly, Holo-Lf (L1294, Sigma Aldrich) was dissolved in 50 mM Tris (hydroxymethyl) aminomethane (pH = 6.8). The protein solution was transferred into a dialysis bag (MWCO = 8,000 Da) and dialyzed against 0.1 M sodium citrate/citric acid 0.1 M NaCl (pH = 3.0) dialysis buffer for 8 h with stirring. The dialysis bag was further dialyzed against Milli-Q water for 48 h. Deionized water was replaced by fresh deionized water every 24 h. Finally, the protein solution was freeze-dried. The obtained Apo-Lf and Holo-Lf were dissolved in the lysis buffer, 40 µg of each protein was loaded onto 12% sodium dodecyl sulfate-polyacrylamide gel (SDS-PAGE), and subsequently incubated with Coomassie brilliant blue fast staining buffer for 15 min. Additionally, Apo-Lf and Holo-Lf were characterized by Fourier transform infrared spectrometer (FTIR).

### Transmission electron microscopic (TEM) images of cells

MDA-MB-231 and MCF-7 cells were seeded in 10 cm^2^ dishes and treated with PBS, erastin (12 μM), Apo-Lf, or Holo-Lf (2 mg/mL). After 24 h, the cells were collected by cell scraper and washed with PBS. After centrifugation, the cells were fixed with 2.5% glutaraldehyde in 0.1 M Sorenson's buffer overnight and post-fixed in 1% osmic acid for 2 h. The cells were dehydrated through an ethanol series (50%-100%) and 100% acetone, embedded in Lx-112 (Ladd Research Industries) and Embed-812 (SPI), and incubated for 24 h at 60 ℃. Subsequently, thin sections were cut on a Leica UC7 ultramicrotome and stained with 2% uranyl acetate and 0.4% lead citrate. Photographs were taken with the Hitachi-7700 transmission electron microscopic (TEM).

### Measurement of reactive oxygen species (ROS) generation

To measure ROS, cells in confocal dishes were fixed with 10% formalin for 15 min after different treatments. ROS probe H2DCFDA (Invitrogen) was dissolved in FBS-free DMEM high glucose culture medium. The cells were then incubated with H2DCFDA solution for 2 h. Finally, cells were incubated with 100 µL 4,6-diamino-2-phenylindole (DAPI) (Invitrogen), and the fluorescence intensity of H2DCFDA was visualized by confocal laser scanning microscopy (CLSM). Additionally, the cells were seeded in 6-well plates for flow cytometry analysis. After incubation with the H2DCFDA solution, the fluorescence intensity of H2DCFDA was analyzed by flow cytometry. 1×10^4^ cells were analyzed per well. The results were normalized to the H2DCFDA intensity of the DMSO group.

### Measurement of glutathione/glutathione disulfide (GSH/GSSG), glutathione peroxidase (GPX) activity, and malondialdehyde (MDA)

GSH, GSSG, and GPX activities were measured using their respective assay kits (Beyotime Institute of Biotechnology). Briefly, cells were cultured in 10 cm^2^ plates overnight and treated with DMSO, 2 μM erastin, Apo-Lf (400 μg/mL), Holo-Lf (400 μg/mL), Apo-Lf + erastin, or Holo-Lf + erastin for 24 h. After treatments, cells were washed with PBS and collected to measure intracellular GSH, GSSG, and GPX activity in accordance with the manufacturer's instructions. To analyze lipid peroxidation after treatments, the MDA concentration in cells was assessed using the MDA assay kit (Beyotime Institute of Biotechnology).

### Measurement of cell membrane permeability

Cell membrane permeability was indicated by the fluorescence intensity of propidium iodide (PI) 38. For PI staining, the cells (2×10^4^) were seeded in confocal dishes. After interventions, the cells were washed with PBS, fixed with 10% formalin for 15 min, and then stained with 10 μL PI solution (1 mg/mL). Finally, the cells were incubated with 100 µL DAPI (Invitrogen) and imaged by CLSM. Moreover, the cells were also collected for flow cytometry analysis. After incubation with PI solution, the fluorescence intensity of PI was analyzed by flow cytometry. 1×10^4^ cells were analyzed per well. The results were normalized to the PI intensity of the DMSO group.

### Western blot analysis

The cells were washed with PBS once and lysed in the lysis buffer (RIPA/protease inhibitor) on ice for 30 min. The cell solution was then centrifuged at 13,000 g for 15 min. The supernatants were collected, and protein concentration was determined according to the BCA protein assay (Beyotime Institute of Biotechnology). Equal amounts of protein were loaded onto the SDS-PAGE gel and subsequently transferred to a polyvinylidene difluoride membrane by electrophoretic transfer. The blots were incubated overnight at 4 °C with the following primary antibodies: bax (1:500, ABclonal), bcl-2 (1:500, ABclonal), ferritin (1:1000, ABclonal), ferroportin (1:1000, Proteintech), GPX-4 (1:1000, Abcam), hypoxia-induced factor 1α (HIF-1α, 1:1000, Cell Signaling Technology), Lf (Lf, 1:1000, ABclonal), SLC7a11 (1:1000, Abcam), transferrin receptor (TfR, 1:1000, Abcam) and β-actin (1:2000, ABclonal). The blots were washed three times, and antigen-antibody complexes were incubated for 1 h at room temperature with Peroxidase Affinity Pure goat anti-mouse or anti-rabbit IgG antibody (1:1,000, Jackson Immuno Research Inc.). Antibody reactivity was detected by chemiluminescence ECL detection systems (EMD Millipore, Darmstadt). The band intensity was normalized by using β-actin density as an internal control. The results were normalized to the control group.

### Quantitative Real-Time PCR (qRT-PCR) analysis

Total RNA was extracted from cells after different treatments using TRIzol reagent (Tiangen). Reverse transcription was performed using 2 µg of each total RNA according to the manufacturer's instructions. The ChamQ^TM^ universal SYBR green PCR master mix (Vazyme) was used for qRT-PCR using a QuantStudio^TM^ 6 flex system (Applied Biosystems). All experiments were carried out using three independent pools. Transcript levels were normalized to GADPH expression. The applied primers are presented in more detail in Supplemental Table 1.

### Measurement of iron content

Total metal concentration was measured using inductively coupled plasma mass spectrometry (ICP-MS, Agilent 7700, Varian). The cells were washed with 5 mL PBS before harvesting with 2 mL of trypsin-EDTA solution. The suspended cells were counted. The cell suspension was centrifuged at 800 rpm for 5 min at room temperature to pellet the cells and subsequently stored at -80 °C until further use. The cells or Lf samples were prepared for ICP-MS analyses as previously described 39. Triple determinations of iron concentration were performed for each sample. Unit conversions from raw ppm values were performed as follows: raw ppm values × dilution factor/cell number.

### Immunofluorescence images

Female BALB/C nude mice bearing MDA-MB-231 tumors (approximately 100 mm^3^) were i.v. injected with PBS, Apo-Lf, or Holo-Lf (4 mg for each mice) for 24 h. The mice were i.v. injected with hypoxia probe pimonidazole hydrochloride (0.6 mg per 10 g body weight) 1.5 h before sacrifice. Subsequently, the tumors were cut into 10 µm sections and fixed with acetone. After washing with PBS twice, the slices were permeabilized in 0.5% Triton X-100 and blocked in 5% BSA and then incubated with anti-pimonidazole antibody (1:100, Hypoxyprobe) or anti-HIF-1α antibody (1:100, CST). After conjugation with corresponding fluorescence secondary antibodies, the slices were observed by CLSM. Also, MDA-MB-231 cells in different groups were incubated with the anti-γ-H2aX monoclonal antibody (1:400, CST) after 4 Gy exposure. The Cy3-conjugated goat anti-rabbit IgG was used as the secondary antibody (1:200, Sangon Biotech). After staining with DAPI, the cells were observed by CLSM. For γ-H2aX foci quantification, the total number of γ-H2aX foci in the nucleus of cells was counted. Analysis of 30 cells in 5 arbitrarily selected microscopic fields was conducted to determine the mean and standard error.

### H_2_O_2_ catalytic ability of Holo-Lf

Intracellular H_2_O_2_ assay was used to evaluate the intracellular H_2_O_2_ catalytic ability of Holo-Lf. MDA-MB-231 cells were cultured under normoxia or hypoxia. The cultured under normoxia were treated with PBS, Apo-Lf, or Holo-Lf overnight. After incubating with 100 μM H_2_O_2_ for 1 h, the cells were collected for further detection. The cells cultured under hypoxia were treated with PBS, Apo-Lf, or Holo-Lf for 24 h under normoxia, followed by a 12 h hypoxia incubation. The cells in both conditions were fixed with 10% formalin and labeled with the H_2_O_2_ fluorescent probe (MAK164, Sigma Aldrich) and α-tubulin monoclonal antibody Alexa Fluor 647 (1:100, MA1-38000-A647, Invitrogen). The formation of hydroxyl radicals (⸳OH) was detected by methylthionine chloride; the absorbance was measured at 666 nm.

### Tumor photoacoustic imaging

Mice bearing MDA-MB-231 tumors were i.v. injected with PBS, Apo-Lf, or Holo-Lf (4 mg). In vivo photoacoustic imaging was performed at different time points (0, 4, 8, 12, and 24 h post-injection) using the oxygen mode with excitation wavelengths of 750 or 850 nm to detect deoxygenated hemoglobin (DO_2_) and oxygenated hemoglobin (HO_2_), respectively. The oxygen saturation level of the tumor area was calculated by sO_2_ = HO_2_/TO_2_, and TO_2_ was calculated by TO_2_ = DO_2_ + HO_2_ 40.

### Biodistribution of Lf in tumor model by CT/SPECT imaging

Holo-Lf was labeled with radionuclide ^125^I (Shanghai GMS Pharmaceutical Co., Ltd.) using the iodogen method 41. In brief, the protein solution (4 mg) was added into the tube pre-coated with iodogen. Subsequently, 200 μCi of ^125^I was added to the protein solution. The mixture was reacted for 30 min at room temperature. Excess ^125^I was removed by centrifugation through Amicon filters (MWCO = 10 kDa) until no substantial radioactivity could be detected in the ultrafiltrate. For CT/SPECT imaging, the obtained ^125^I-Lf were i.v. injected into mice bearing MDA-MB-231 tumors and imaged by in vivo animal CT/SPECT imaging system. Finally, the mice were sacrificed, and major organs (heart, liver, spleen, lung, kidney, brain, and tumor) were collected for biodistribution analysis.

### Anti-tumor study

Sixteen mice bearing MDA-MB-231 tumors (approximately 100 mm^3^) were randomly divided into four groups and treated with PBS, 4 Gy, Apo-Lf+4 Gy, or Holo-Lf+4 Gy,. The mice were i.v. injected with Apo-Lf or Holo-Lf every 2 days. The Lf dosage was 2 mg for each mouse. The tumors were measured with a digital caliper and the size of the tumor was calculated using the following equation: Volume = Length × (Width)^2^/2. Tumor volumes were recorded with a caliper every 2 days. The mice with a tumor volume above 1000 mm^3^ were treated as dead. After 16 days of treatment, the mice were sacrificed. Major organs (heart, liver, spleen, lung, and kidney) in each group were collected for H&E staining. Tumors were collected for GPX-4 immunohistochemical staining.

### Statistical analyses

Statistical analyses were performed using the one-way ANOVA test and calculated with SPSS 26.0 software. All experiments were conducted in triplicate and data were presented as mean ± SD. A *P* value of less than 0.05 was considered as statistically significant.

## Results

### Preparation of Apo-Lf

A low iron-saturated form of human Lf (Apo-Lf) was first prepared to investigate the role of Lf iron in ferroptosis. Each Lf molecule has two iron-binding sites. When exposed to the acidic environment, the iron content of Lf was gradually released from its binding sites 42, 43. We dialyzed Holo-Lf against sodium citrate/citric acid solution (pH = 3) for 8 h (Figure S1A), following which about 79.5% of iron was released from Holo-Lf (Figure S1B). Coomassie blue staining and Western blotting revealed that Apo-Lf and Holo-Lf had the same molecular weight (≈80 kDa) and the ability to bind to the anti-Lf antibody (Figure S1C-D). Apo-Lf and Holo-Lf also showed similar characteristics by FTIR spectrometry (Figure S1E). These results indicated no substantive changes in the structure of Apo-Lf after dialysis.

### Holo-Lf induced ferroptosis of MDA-MB-231 cells

**Figure 1:**
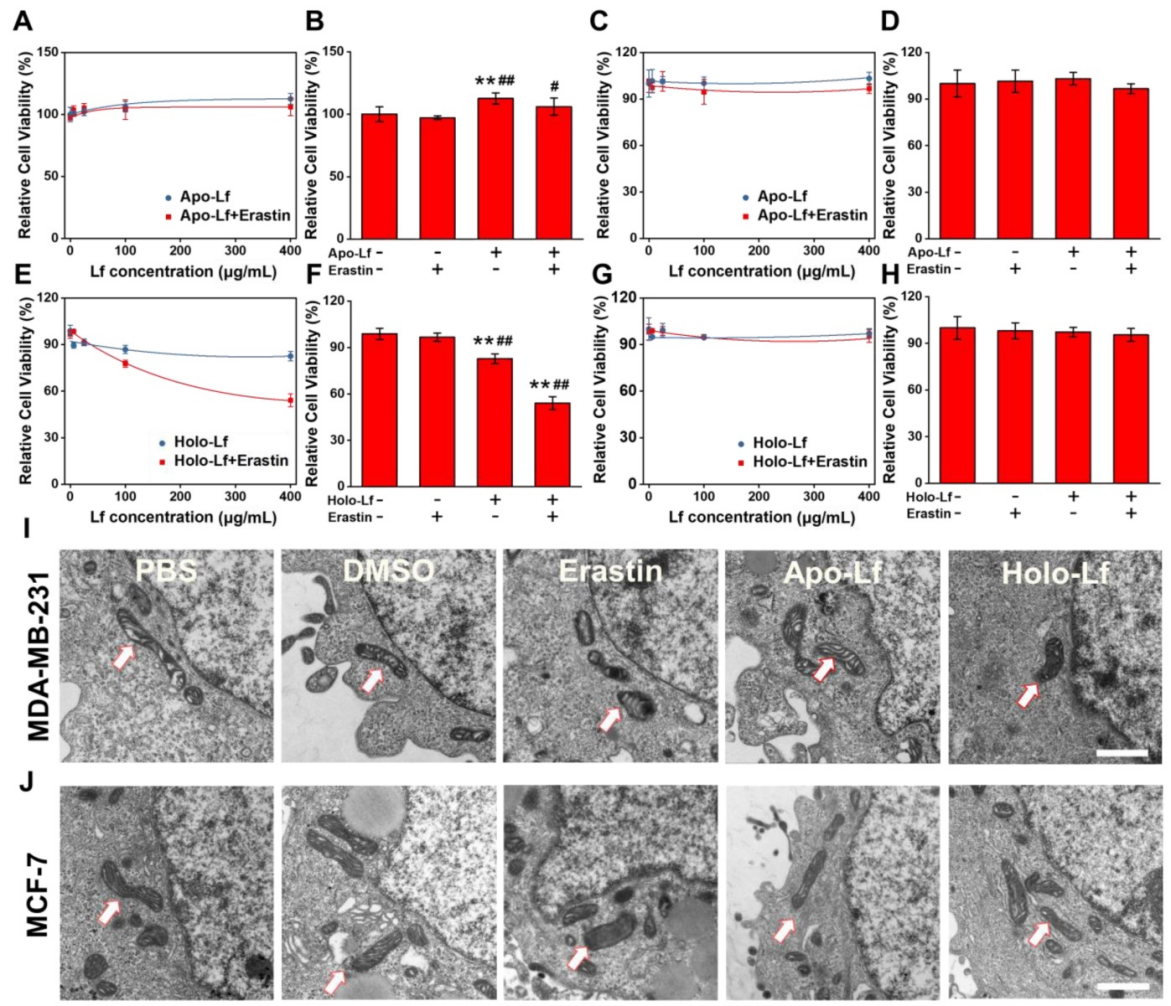
Influence of Apo-Lf and Holo-Lf on cell viability and morphology: (A-H) Relative cell viability of MDA-MB-231 (A-B, E-F) and MCF-7 cells (C-D, G-H) incubated with Apo-Lf, Holo-Lf (400 μg/mL) with or without erastin (2 μM) (n = 3). (I-J) TEM images of MDA-MB-231 (I) and MCF-7 cells (J) incubated with DMSO, erastin (12 μM), Apo-Lf (2 mg/mL) and Holo-Lf (2 mg/mL). Single white arrowheads show shrunken mitochondria. At least 10 cells in each group were examined. Scale bar, 1 μm. Data are shown as mean ± SD. ^*^Compared with control group *P* < 0.05. ^**^Compared with control group *P* < 0.01. ^#^Compared with erastin group *P* < 0.05. ^##^Compared with erastin group *P* < 0.01.

The TNBC cell line MDA-MB-231 and non-TNBC cell line MCF-7 were employed to explore the different roles of Apo-Lf and Holo-Lf in ferroptosis. Holo-Lf remarkably inhibited the cell viability of MDA-MB-231 cells, and the effect was further intensified in combination with erastin, an agonist of ferroptosis (Figure 1E-F). However, Holo-Lf cytotoxicity to MDA-MB-231 cells was attenuated by ferroptosis inhibitor ferrostatin-1 (Fer-1) (Figure S2A). By contrast, Apo-Lf was not cytotoxic to MDA-MB-231 cells and even increased the cell viability (Figure 1A-B). Apo-Lf and Holo-Lf did not influence the viability of MCF-7 cells (Figure 1C-D, G-H). For transferrin (Tf), both Apo-Tf and Holo-Tf inhibited the cytotoxicity of erastin to MDA-MB-231 cells (Figure S2B). Unlike the morphology of autophagy, apoptosis, or necrosis, ferroptosis exhibits a single distinctive morphological feature in mitochondria 19. Cell samples were prepared for TEM to determine whether Holo-Lf-induced cell death shared any morphological similarities with ferroptosis. After treatment with erastin, MDA-MB-231 cells exhibited shrunken mitochondria with increased membrane density and reduced mitochondrial cristae. Compared with DMSO-treated cells, a similar morphology was also observed in MDA-MB-231 cells treated with Holo-Lf but not Apo-Lf (Figure 1I). For MCF-7 cells, the typical ferroptosis mitochondrion morphology was induced by erastin treatment but was rarely observed in cells treated with Holo-Lf (Figure 1J). These results prompted the investigation of the relationship between Holo-Lf and ferroptosis.

**Figure 2:**
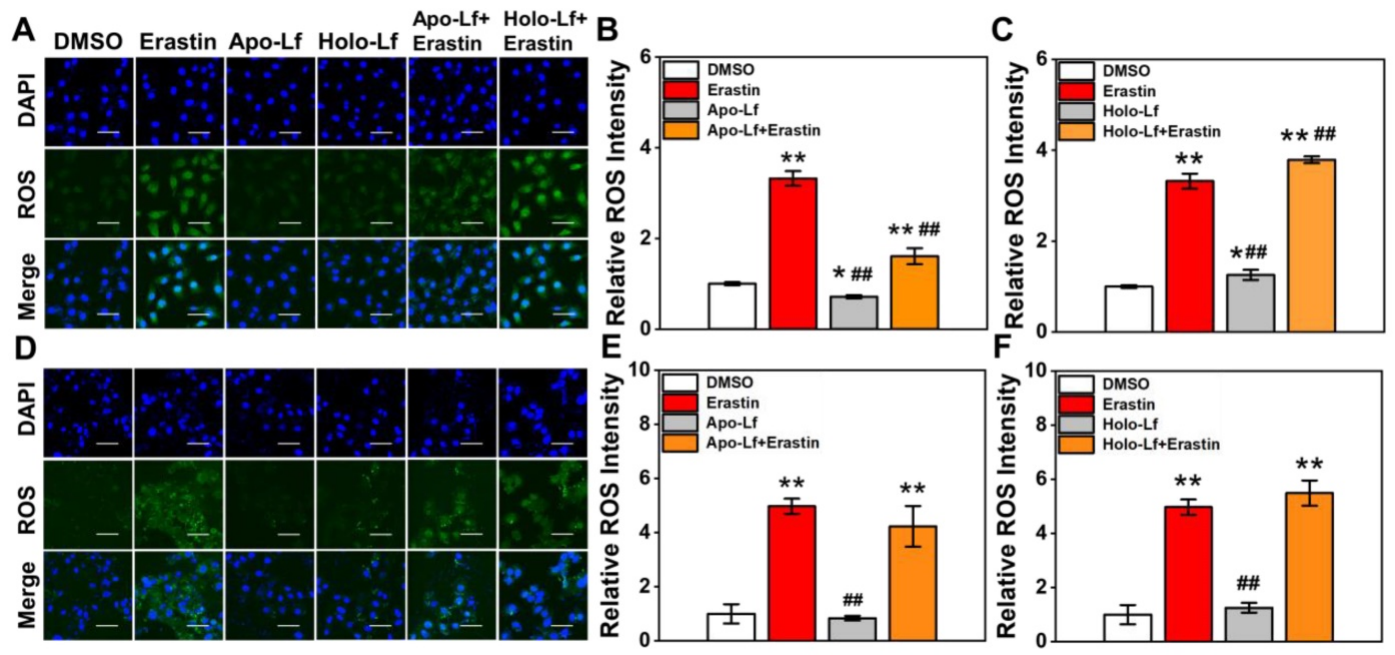
ROS fluorescence images and relative ROS fluorescence intensities of MDA-MB-231(A-C) and MCF-7 cells (D-F) incubated with Apo-Lf, Holo-Lf (400 μg/mL) with or without erastin (2 μM) (n = 3). Scale bar, 100 μm. Data are shown as mean ± SD. ^*^Compared with DMSO group *P* < 0.05. ^**^Compared with DMSO group* P* < 0.01. ^##^Compared with erastin group *P* < 0.01.

ROS accumulation was a critical modulator of ferroptosis. We observed that Holo-Lf significantly increased ROS generation in MDA-MB-231 cells in a dose-dependent manner (Figure 2A, C & Figure S2C). When combined with erastin, ROS generation in MDA-MB-231 cells was further promoted (Figure 2A, C). On the contrary, Apo-Lf decreased ROS-generation and inhibited the ROS-promoting effect of erastin in MDA-MB-231 cells (Figure 2A-B). For MCF-7 cells, both Apo-Lf and Holo-Lf had no significant influence on ROS generation (Figure 2D-F).

**Figure 3:**
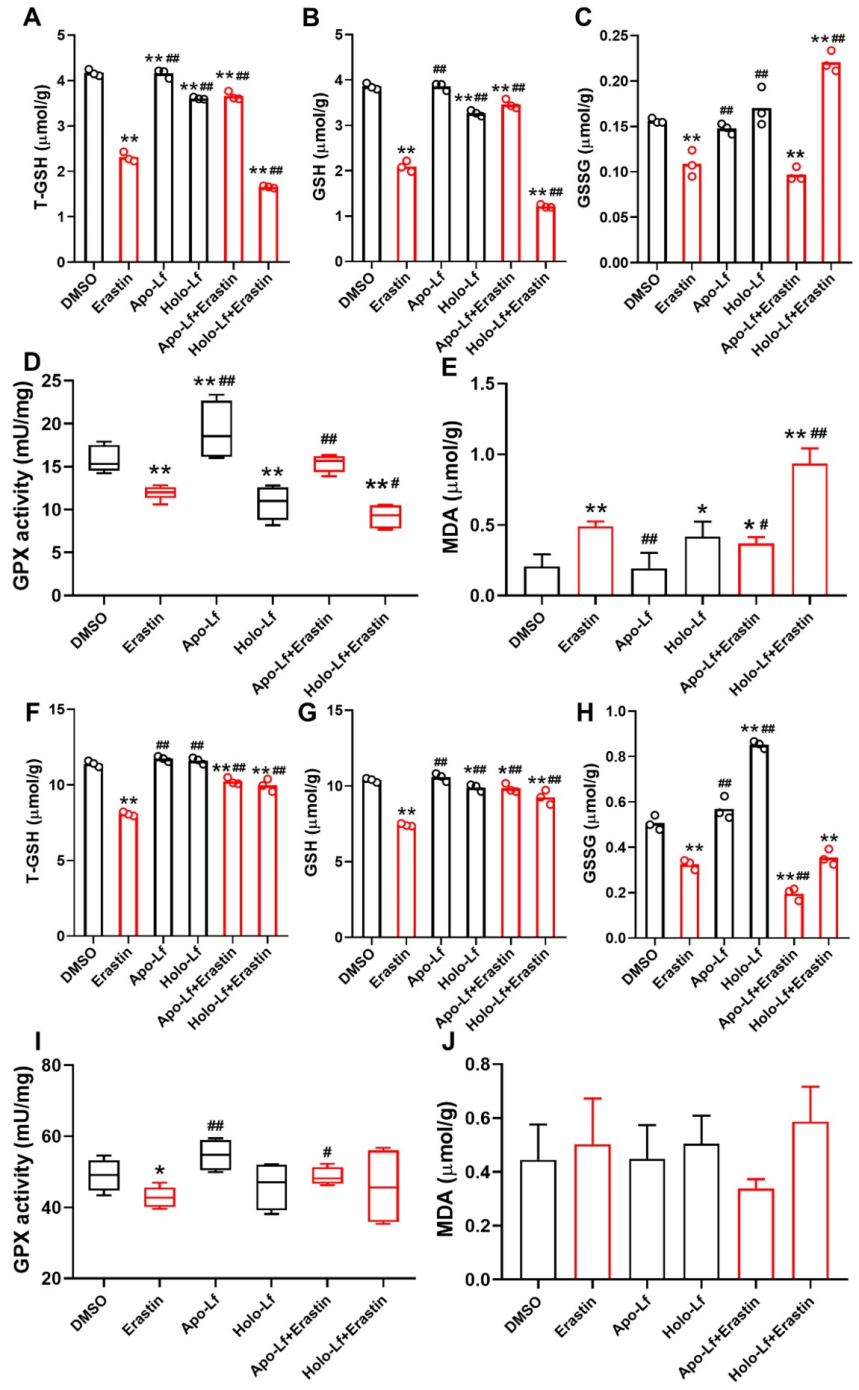
Total glutathione (T-GSH), reduced glutathione (GSH), oxidized glutathione (GSSG), glutathione peroxidase (GPX) activity and malondialdehyde (MDA) of MDA-MB-231 (A-E) and MCF-7 cells (F-J) incubated with DMSO, Apo-Lf (400 μg/mL), Holo-Lf (400 μg/mL) with or without erastin (2 μM) (n = 3). Data are shown as mean ± SD. ^*^Compared with DMSO group *P* < 0.05. ^**^Compared with DMSO group *P* < 0.01. ^#^Compared with erastin group *P* < 0.05. ^##^Compared with erastin group *P* < 0.01.

Lipid peroxidation end product MDA plays a central role in the induction of ferroptosis 44. GPX reduces lipid hydroperoxides to protect organisms from ROS-induced oxidative stress damage at the expense of oxidizing reduced glutathione (GSH) to its disulfide form (GSSG) 45. We found that erastin significantly decreased the concentration of total GSH (T-GSH), GSH, and GSSG and the activity of GPX in MCF-7 and MDA-MB-231 cells but only increased the generation of MDA in MDA-MB-231 cells. Like erastin, Holo-Lf reduced the concentration of T-GSH, GSH, and the activity of GPX in MDA-MB-231 cells only, and this effect was further promoted by erastin. However, when combined with erastin, Apo-L exhibited an opposite influence on the indicators mentioned above in MDA-MB-231 cells (Figure 3A-E). In MCF-7 cells, both Apo-Lf and Holo-Lf failed to sustainably reverse or enhance the effect of erastin on the concentration of T-GSH, GSH, and MDA (Figure 3F-J).

**Figure 4:**
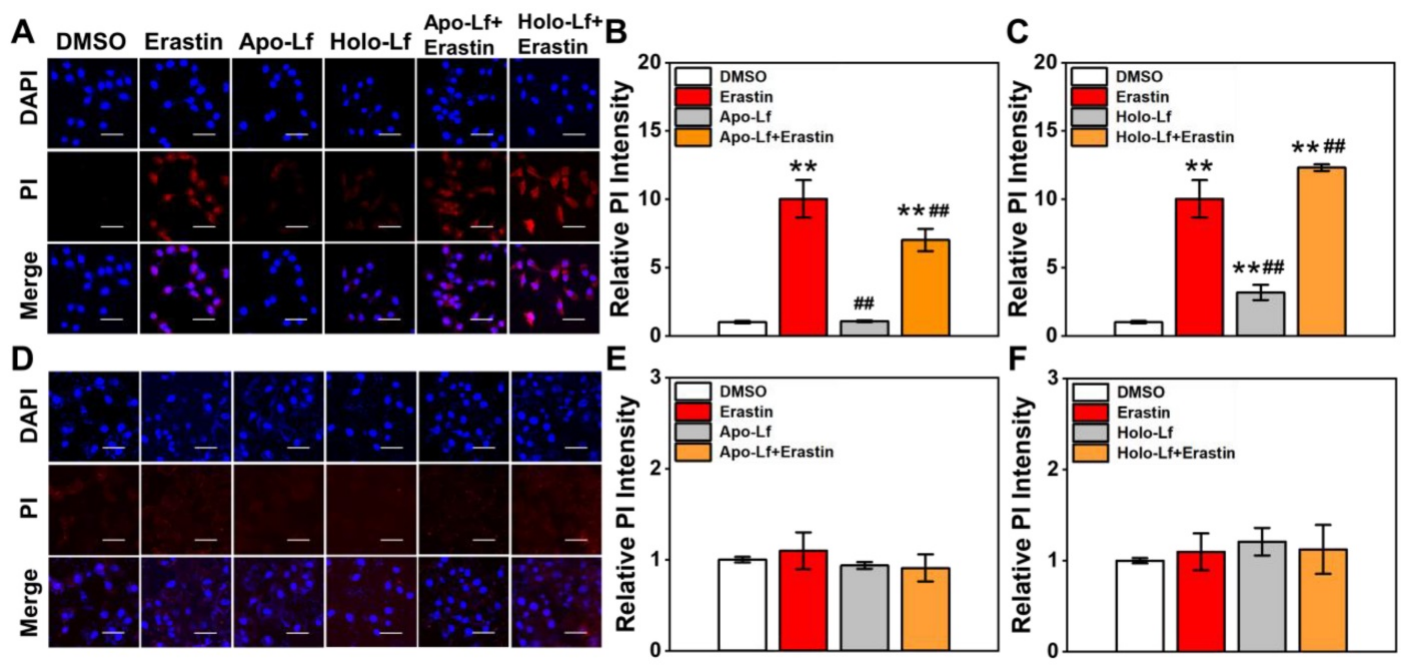
Influence of Apo-Lf and Holo-Lf on cell membrane permeability: Propidium iodide (PI) fluorescence images and relative PI fluorescence intensities of MDA-MB-231(A-C) and MCF-7 cells (D-F) incubated with Apo-Lf (400 μg/mL), Holo-Lf (400 μg/mL) with or without erastin (2 μM) (n = 3). Scale bar, 100 μm. Data are shown as mean ± SD or median (interquartile range). ^**^Compared with DMSO group *P* < 0.01. ^##^Compared with erastin group *P* < 0.01.

An increased cell membrane permeability reflects an elevated accumulation of lipid peroxidation products in the cell membrane. Similar to MDA results, Holo-Lf significantly increased cell membrane permeability in MDA-MB-231 cells that was further enhanced when combined with erastin (Figure 4A, C). However, when combined with erastin, Apo-Lf considerably decreased cell membrane permeability in MDA-MB-231 cells (Figure 4A, B). For MCF-7 cells, both Apo-Lf and Holo-Lf failed to induce a significant influence on cell membrane permeability (Figure 4D-F).

**Figure 5:**
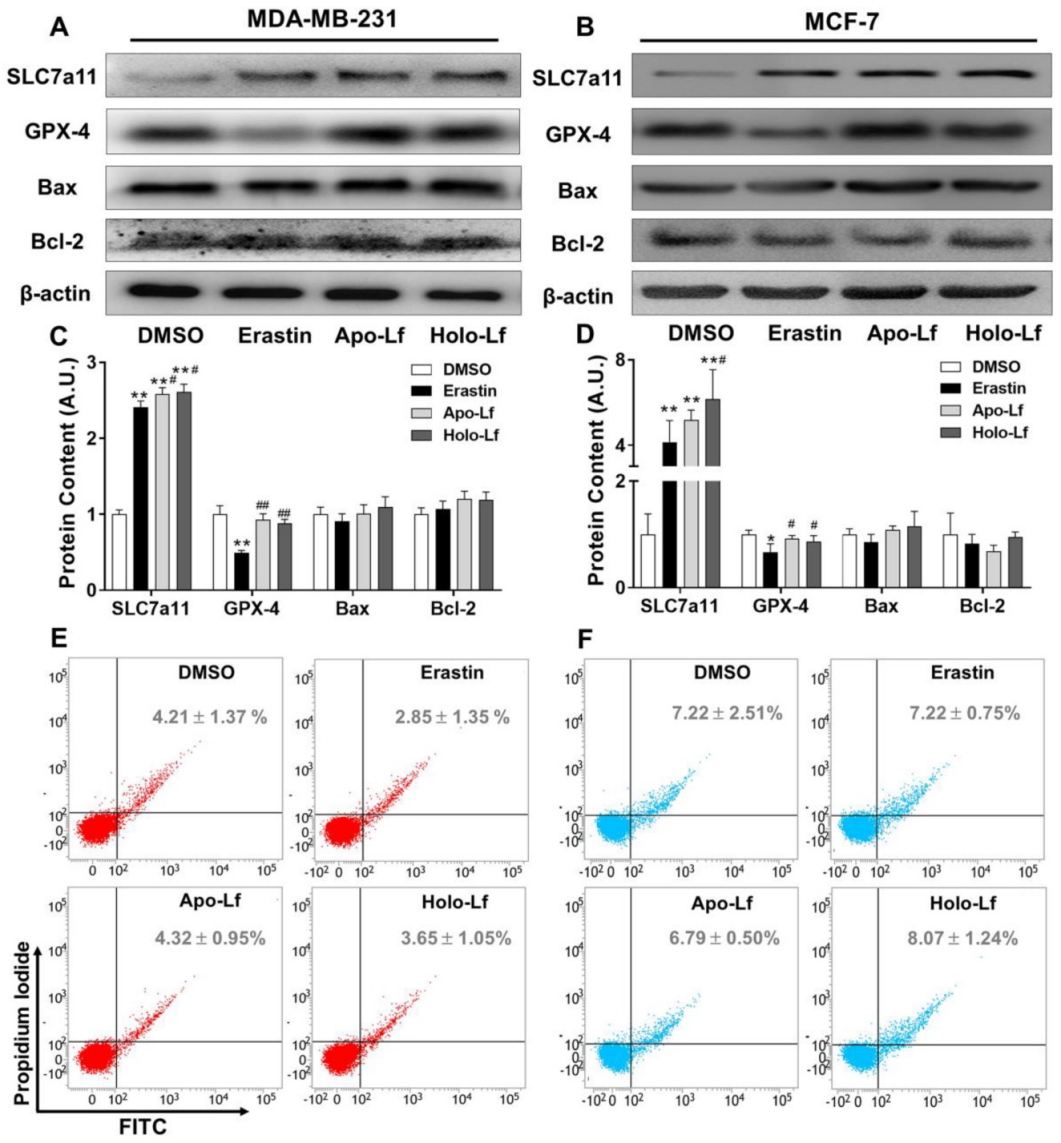
Influence of Apo-Lf and Holo-Lf on the expression of SLC7a11, GPX-4, and apoptosis: (A-D) Western blot analysis of SLC7a11, GPX-4, Bax, and Bcl-2 in MDA-MB-231 (A/C) and MCF-7 cells (B/D) incubated with DMSO, erastin (2 μM), Apo-Lf (400 μg/mL) and Holo-Lf (400 μg/mL). The total grey intensity of each protein was normalized to the grey intensity of an appropriate protein in cells treated with DMSO. (E/F) Percentage of total apoptosis in MDA-MB-231 and MCF-7 cells (n = 3). Data are shown as mean ± SD. ^*^Compared with DMSO group *P* < 0.05. ^**^Compared with DMSO group *P* < 0.01. ^#^Compared with erastin group *P* < 0.05. ^##^Compared with erastin group *P* < 0.01.

SLC7a11 is a subunit of the cystine/glutamate antiporter-system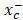 and is responsible for importing cystine into cells for GSH biosynthesis and maintaining the antioxidant activity of GPX-4 46, 47. As an inhibitor of the system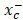, erastin upregulated the transcription and translation of SLC7a11 but downregulated the transcription and translation of GPX-4 in MCF-7 and MDA-MB-231 cells (Figure 5A-D & Figure S3A-B). Apo-Lf and Holo-Lf significantly upregulated the protein and gene levels of SLC7a11 in both cells but did not influence the GPX-4 expression (Figure 5A-D & Figure S3A-B). This finding indicated that Apo-Lf may inhibit the ferroptosis in MDA-MB-231 cells by promoting cystine uptake and GSH synthesis.

To further determine whether Holo-Lf induced apoptosis of MDA-MB-231 cells under our experimental conditions, the expression of Bax and Bcl-2 and the percentage of total apoptotic cells in MDA-MB-231 and MCF-7 cells after different treatments were analyzed. Although Lf was previously reported to induce apoptosis in cancer cells 11, 48, we observed no influence of Holo-Lf on the transcription and translation of Bax and Bcl-2 and the percentage of total apoptotic cells (Figure 5 & Figure S3A-B). Apo-Lf and Holo-Lf did not influence the apoptosis of MDA-MB-231 and MCF-7 cells under our experimental conditions. Collectively, our findings confirmed the superior anticancer effect of Holo-Lf by inducing/causing ferroptosis in MDA-MB-231 cells.

### MCF-7 cells were resistant to ferroptosis

**Figure 6:**
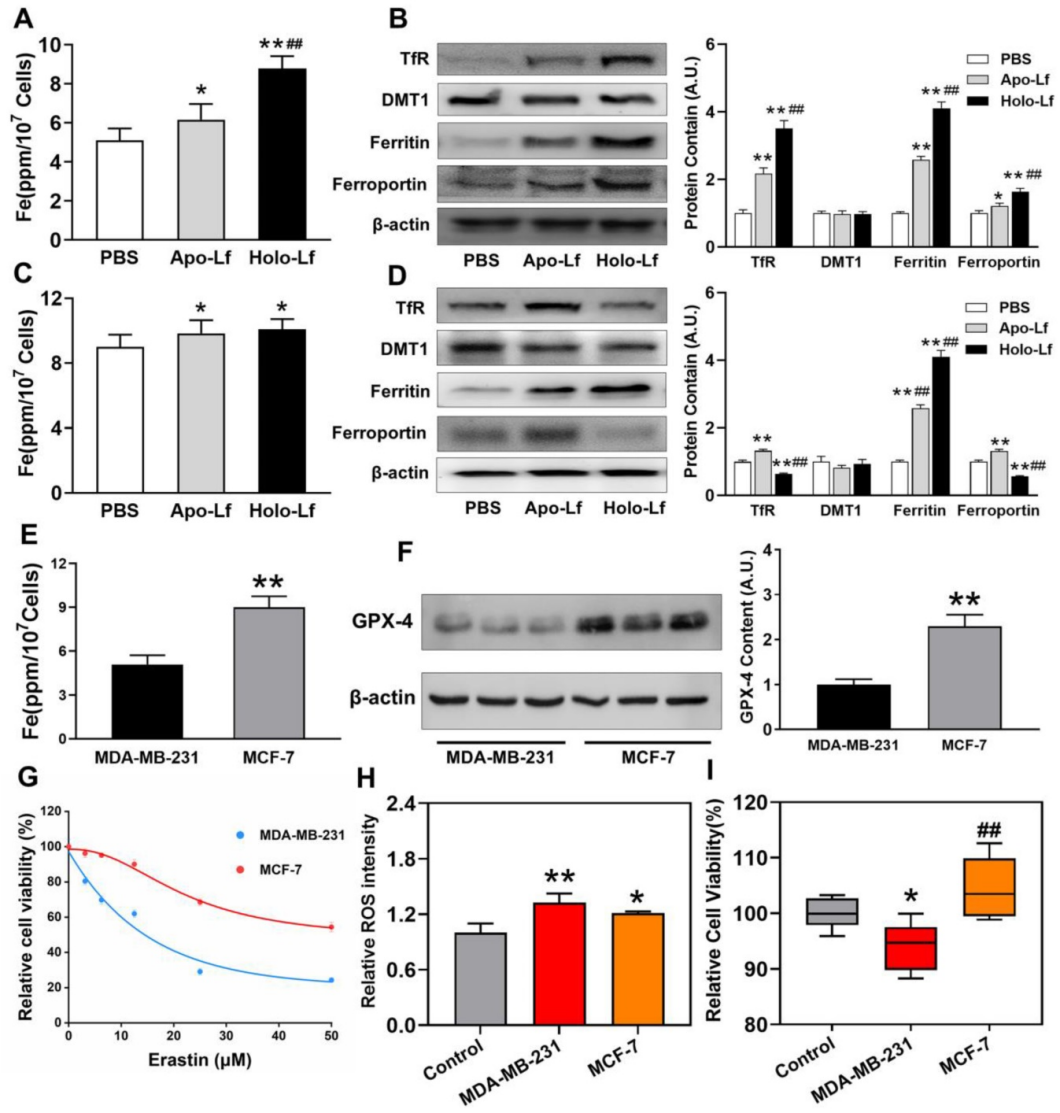
Different iron homeostatic regulation and redox balance capacity between MDA-MB-231 and MCF-7 cells: (A-D) Intracellular iron measurement and Western blot analysis of TfR, ferritin, and ferroportin of MDA-MB-231 (A-B) and MCF-7 (C-D) cells incubated with PBS, Apo-Lf, and Holo-Lf (400 μg/mL) (n = 3). ^*^Compared with PBS group *P* < 0.05. ^**^Compared with PBS group *P* < 0.01. ^##^Compared with Apo-Lf group *P* < 0.01. (E) Total iron content of MDA-MB-231 and MCF-7 cells. (F) Western blot analysis of GPX-4 in MDA-MB-231 and MCF-7 cells (n = 3). ^**^Compared with MDA-MB-231 cells *P* < 0.01. (G) Relative cell viability of MDA-MB-231 and MCF-7 cells incubated with various concentrations of erastin (n = 6). (H-I) Relative ROS (n = 3) and cell viability (n = 6) of MDA-MB-231 and MCF-7 cells incubated with FeCl_3_. ^*^Compared with control group *P* < 0.05. ^**^Compared with control group *P* < 0.01. ^##^Compared with MDA-MB-231 cells *P* < 0.01. Total grey intensity of each protein was normalized to the grey intensity of an appropriate protein in cells treated with PBS or MDA-MB-231 cells. Data are shown as mean±SD.

Despite the unique role of Holo-Lf on ferroptosis, different ferroptosis sensitivities were observed between MDA-MB-231 and MCF-7 cells. Iron homeostasis regulation after different treatments was monitored to decipher the underlying mechanism. Intracellular iron is involved in Fenton chemistry, elevates the oxidative stress of cells and organisms 49 and iron overload might induce ferroptosis. We found that the total iron content of MDA-MB-231 cells was lower than MCF-7 cells (Figure 6E). Apo-Lf significantly increased the total iron content in both cells that might be attributed to the remaining iron in Apo-Lf after dialysis. Compared with Apo-Lf, Holo-Lf further increased the total iron content in MDA-MB-231 cells but not in MCF-7 cells (Figure 6A, C). We measured the expression of iron metabolism-related proteins and found that Holo-Lf upregulated TfR, ferritin, and ferroportin in MDA-MB-231 cells but downregulated TfR and ferroportin in MCF-7 cells (Figure 6B, D). When both cells were incubated with labile iron-FeCl_3,_ ROS generation was increased in both cells, but the cell viability was inhibited only in MDA-MB-231 cells (Figure 6H-I). Because of the difference in iron homeostasis regulation, MDA-MD-231 cells had a higher risk of iron overloading than MCF-7 cells. Moreover, the protein and mRNA levels of GPX-4 in MCF-7 cells were higher than in MDA-MB-231 cells (Figure 6F & Figure S3C). After incubation with various erastin concentrations, a higher percentage of MDA-MB-231 cells died (Figure 6G), indicating the superior redox balance capacity of MCF-7 cells compared to MDA-MB-231 cells. In summary, these findings explained the higher sensitivity of MDA-MB-231 cells to Holo-Lf-induced ferroptosis than MCF-7 cells.

### Holo-Lf ameliorated the hypoxia microenvironment of MDA-MB-231 cells and tumors

**Figure 7:**
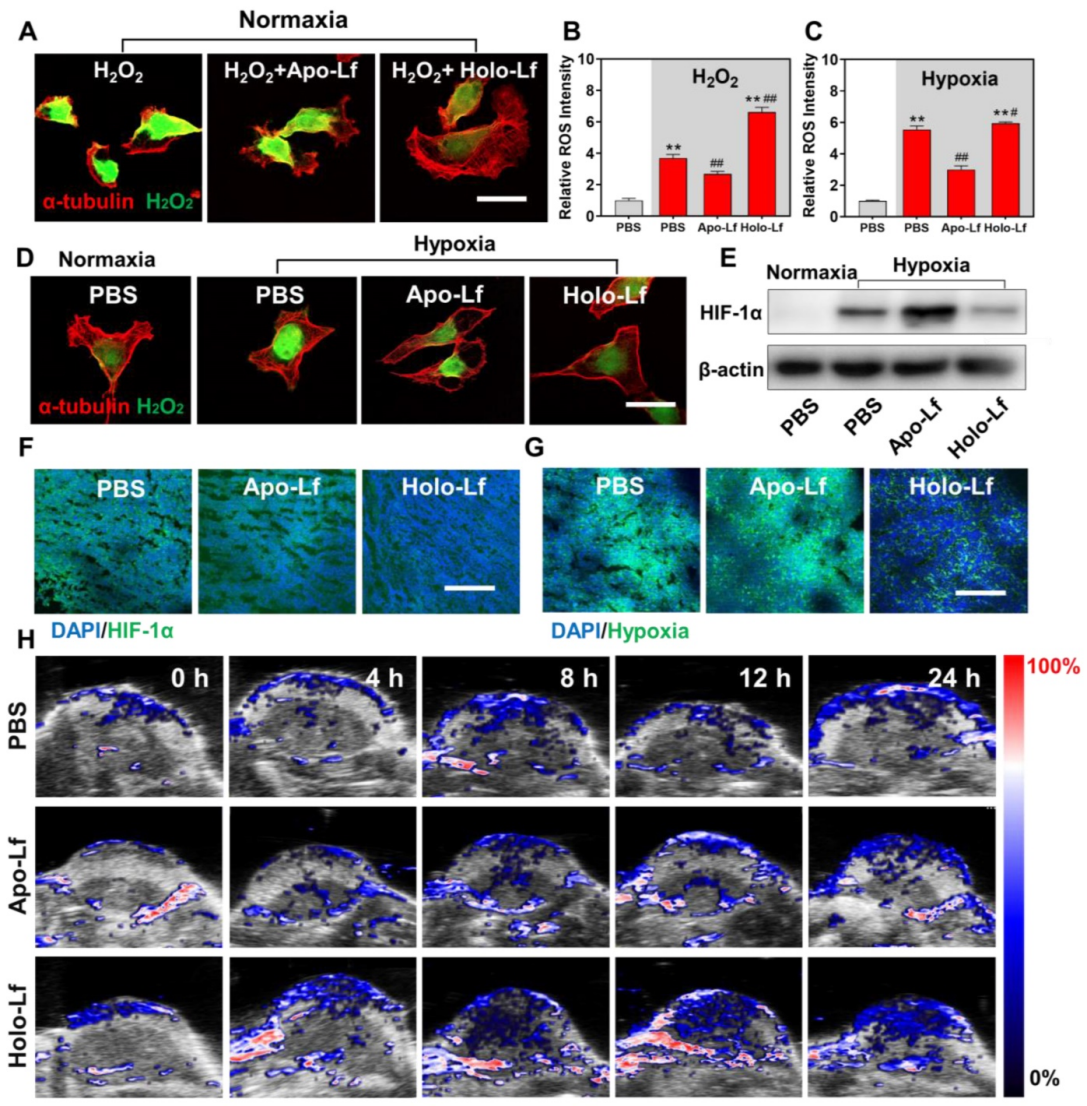
Influence of Apo-Lf and Holo-Lf on hypoxia: (A) Intracellular H_2_O_2_ CLSM images of MDA-MB-231 cells treated with H_2_O_2_, Apo-Lf+H_2_O_2_, and Holo-Lf+H_2_O_2_ under normoxia. Scale bar, 30 μm. (B) Relative ROS intensity of MDA-MB-231 cells under normoxia (n = 3). (C) Relative ROS intensity of MDA-MB-231 cells incubated under normoxia and hypoxia (n = 3). (D) Intracellular H_2_O_2_ CLSM images of MDA-MB-231 cells incubated with PBS under normoxia, and cells incubated with PBS, Apo-Lf, and Holo-Lf under hypoxia. Scale bar, 30 μm. (E) HIF-1α images of MDA-MB-231 cells incubated with PBS under normoxia, and cells incubated with PBS, Apo-Lf, and Holo-Lf under hypoxia. (Cells incubated in hypoxia for 12 h). (F-G) HIF-1α and hypoxia images of tumor slices collected from MDA-MB-231 tumor-bearing mice i.v. injected with PBS, Apo-Lf, and Holo-Lf (4 mg for each mouse). Scale bar, 200 μm. (H) In vivo photoacoustic imaging of MDA-MB-231 tumor-bearing mice injected with PBS, Apo-Lf, and Holo-Lf at different time points. Data are shown as mean ± SD. ^**^Compared with cells cultured under normoxia or PBS group *P* < 0.01. ^#^Compared with cells cultured under hypoxia or PBS group *P* < 0.05. ^##^Compared with cells cultured under hypoxia or PBS group *P* < 0.01.

Since MCF-7 cells were resistant to oxidative stress, we explored the Lf effect on hypoxia and radiotherapy efficacy in MDA-MB-231 cells only. H_2_O_2_ concentration is usually high in the tumor microenvironment and hypoxic cells. Converting intracellular H_2_O_2_ into O_2_ through Fenton reaction has been shown to be an effective way to improve hypoxia 50, 51. In our study, Holo-Lf increased the iron level in MDA-MD-231 cells, but it was unclear whether Holo-Lf could ameliorate hypoxia microenvironment by catalyzing the decomposition of H_2_O_2_. Therefore, we incubated MDA-MB-231 cells in the H_2_O_2_ culture medium to imitate the high H_2_O_2_ concentration of cells in hypoxia. Holo-Lf was found to increase the ⸳OH generation in vitro (Figure S4A). Following treatment with Holo-Lf, the intracellular H_2_O_2_ was decreased, but ROS was increased (Figure 7A-B). Subsequently, when MDA-MB-231 cells were cultured in the hypoxia chamber, Holo-Lf decomposed a significant amount of H_2_O_2_ and elevated the ROS level of hypoxia in MDA-MB-231 cells (Figure 7C-D).

HIF-1α is an important indicator of hypoxia. It is upregulated in hypoxic condition and quickly degraded by the von Hippel-Lindau (VHL) protein under normoxia 52. We observed that HIF-1α was downregulated by Holo-Lf, but further elevated by Apo-Lf in hypoxic MDA-MB-231 cells (Figure 7E & Figure S4B). The expression of HIF-1α was also detected in solid tumors. Consistent with the results observed in hypoxic cells, Holo-Lf significantly decreased the percentages of HIF-1α-positive and hypoxia-positive areas in the MDA-MB-231 tumor (Figure 7F-G & Figure S4D-E). Furthermore, the oxygenated hemoglobin concentration in the central region of the tumor was increased after i.v. injection of Holo-Lf (Figure 7H & Figure S4C). However, Apo-Lf had no significant effect on HIF-1α expression and oxygenated hemoglobin concentration in solid tumors. These observations suggested that Holo-Lf could ameliorate the tumor hypoxia microenvironment by catalyzing H_2_O_2_ decomposition.

### Holo-Lf sensitized MDA-MB-231 cells to radiotherapy

**Figure 8:**
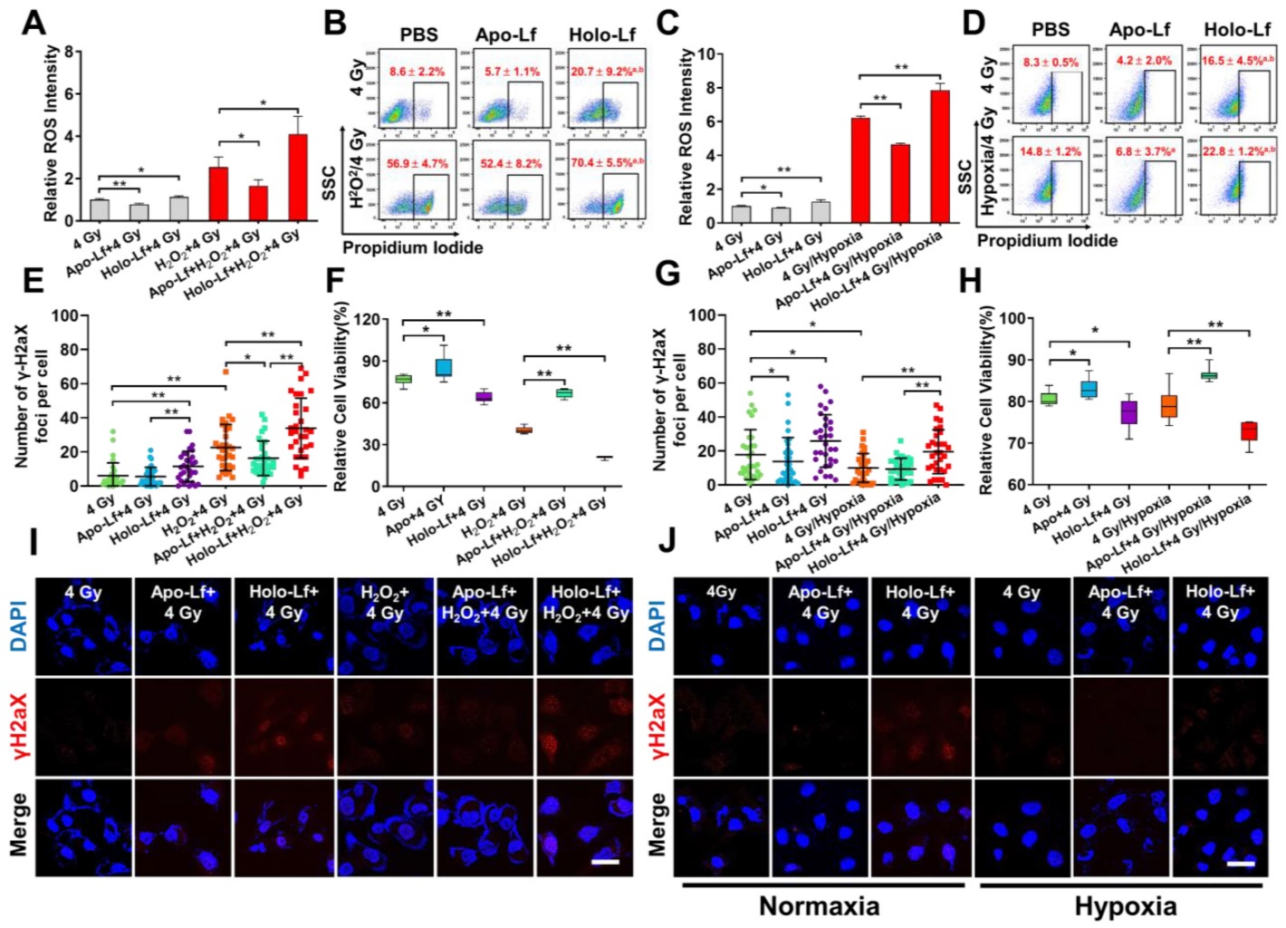
Influence of Apo-Lf and Holo-Lf on the radiosensitivity of MDA-MB-231 cells: (A/C) Relative ROS fluorescence intensity of MDA-MB-231cells after different treatments (n = 3). (B/D) Percentage of positive PI-stained cells with different treatments (Cells with PI intensity above 10^3^ were treated as positive PI-stained cells) (n = 3). (E/G) Number of γ-H2aX foci in MDA-MB-231 cells after different treatments (n = 30). (F/H) Relative cell viability of cells after different treatments. Cell viability was measured 24 h after radiotherapy (n = 10). (I/J) γH2aX fluorescence imaging of cells after different treatments (Apo-Lf and Holo-Lf: 400 μg/mL; H_2_O_2_: 200 μM; cells were incubated under hypoxia for 12 h). Scale bar, 100 μm. Data are shown as mean ± SD or median (interquartile range). ^a^Compared with PBS group* P* < 0.05. ^b^Compared with Apo-Lf group* P* < 0.05. ^*^P < 0.05.^ **^P < 0.01.

The influence of Lf on radiotherapy efficacy was monitored in MDA-MB-231 cells incubated with or without H_2_O_2_. Holo-Lf significantly increased ROS generation and percentage of positive PI staining in cells without H_2_O_2_ incubation following exposure to 4 Gy radiation (Figure 8A-B). A higher number of nuclear γH2aX foci was also observed (Figure 8E, I) and a higher percentage of MDA-MB-231 cells was inhibited by Holo-Lf one day after radiotherapy (Figure 8F). Compared with cells incubated without H_2_O_2_, those incubated with H_2_O_2_ had substantially elevated ROS generation. More significantly, Holo-Lf substantially increased ROS generation and the percentage of positive PI staining of cells (Figure 8A-B). As revealed by the number of γH2aX foci, Holo-Lf induced the most serious DNA damage to MDA-MB-231 cells, remarkably inhibiting the cell viability (Figure 8E-F, I). However, Apo-Lf played an opposite role in ROS generation, DNA damage, and cell viability in MDA-MB-231 cells with or without H_2_O_2_ incubation.

The influence of Apo-Lf and Holo-Lf on the radiotherapy efficacy was verified in MDA-MB-231 cells incubated in normoxia and hypoxia. ROS was quickly generated by anaerobic glycolysis in the hypoxic culture environment, and the percentage of cells with positive PI staining was also increased (Figure 8C-D). However, the radiation-induced DNA damage to MDA-MB-231 cells was significantly inhibited in the hypoxic environment (Figure 8G, J). Holo-Lf treatment increased the percentage of positive PI-stained cells after 4 Gy radiation (Figure 8D). The number of γH2aX foci was also increased, compared to the cells cultured in normoxia (Figure 8G, J). Eventually, the viability of MDA-MB-231 cells was further inhibited (Figure 8H). By contrast, Apo-Lf decreased ROS generation, the percentage of positive PI-stained cells, and viability of MDA-MB-231 cells cultured in the hypoxia environment. These data suggested that Holo-Lf could sensitize MDA-MB-231 cells to radiotherapy in hypoxic condition.

### Holo-Lf enhanced the efficacy of radiotherapy in MDA-MB-231 tumors

**Figure 9:**
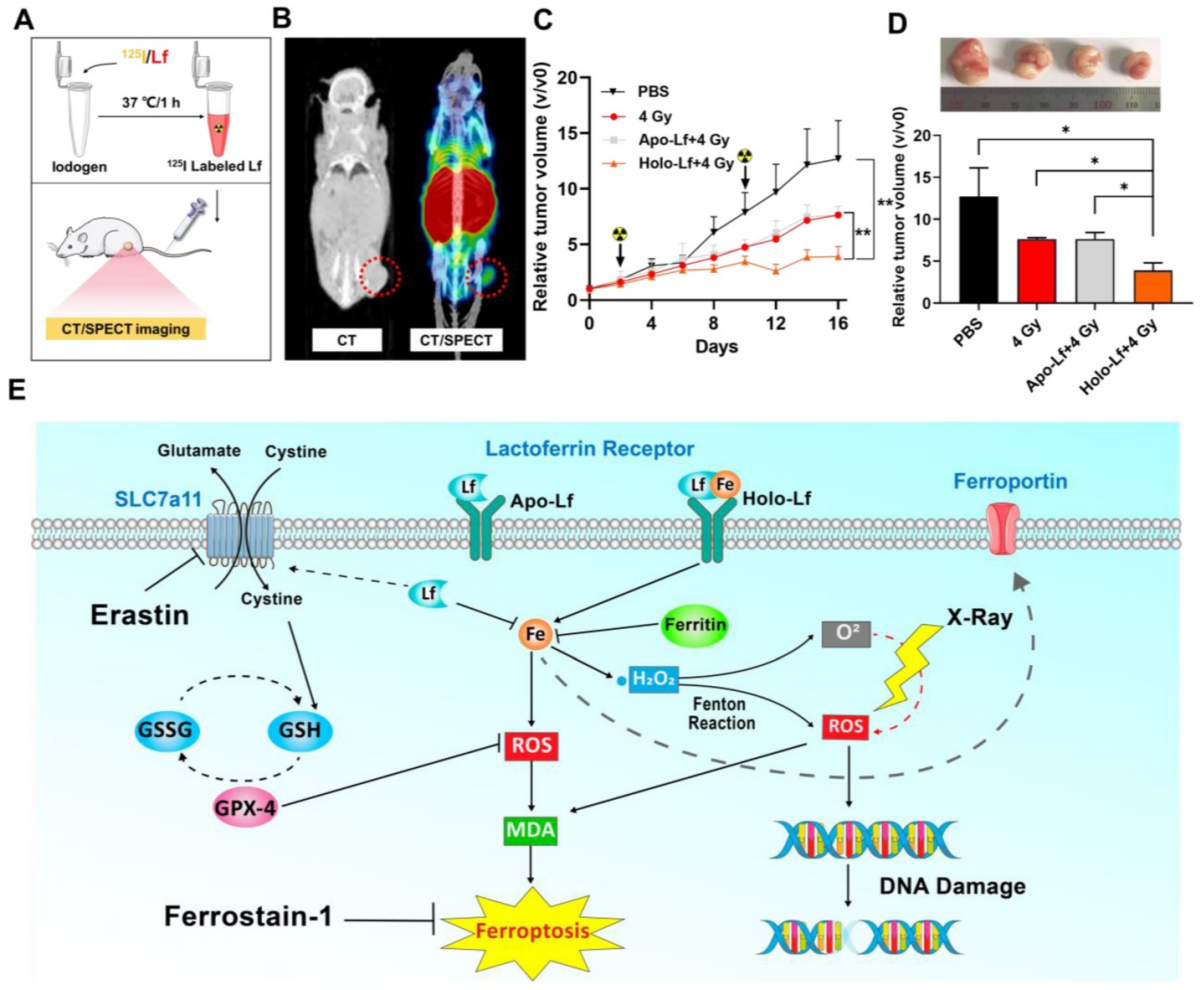
Influence of Apo-Lf and Holo-Lf on radiotherapy efficacy in MDA-MB-231 tumors: (A) Preparation of ^125^I-labeled Holo-Lf. (B) CT/SPECT imaging of mouse i.v. injected with ^125^I-Holo-Lf. (C) Relative tumor volume (V/V0) changing curves of different treatment groups (PBS, 4 Gy, Apo-Lf+4 Gy, and Holo-Lf+4 Gy) (n = 4). Data are shown as mean ± SD. ^*^P < 0.05. (D) Digital photograph of tumors and final tumor volume in four groups (n = 4). (E) Schematic illustration showing the different roles of Apo-Lf and Holo-Lf in ferroptosis and radiotherapy. Apo-Lf decreased ROS generation by decreasing labile iron and upregulating SLC7a11 expression and attenuated erastin-induced ferroptosis and sensitivity of MDA-MB-231 cells to radiotherapy. However, Holo-Lf released its bound iron to increase ROS generation and ameliorate the hypoxic environment of the tumor. As a result, Holo-Lf promoted ferroptosis and sensitivity of MDA-MB-231 cells and tumors to radiotherapy. ^*^P < 0.05.

To assess the influence of Holo-Lf on the radiotherapy efficacy of the MDA-MB-231 tumor, first an in vivo biodistribution experiment was performed. After labeling with ^125^I, the in vivo biodistribution of Holo-Lf was monitored by CT/SPECT imaging. A significant amount of Lf immediately accumulated in the tumor site after injection (Figure 9A-B & Figure S5A), explaining the potential role of Holo-Lf in the tumor site. Subsequently, an anti-tumor experiment was conducted. MDA-MB-231 tumor growth was significantly repressed by 4 Gy radiation and was further inhibited when combined with Holo-Lf (Figure 9C-D). The survival rate in the Holo-Lf+4 Gy group was also higher than the 4 Gy group (75% vs 50%) (Figure S5B). There was no difference in the body weights between the two groups (Figure S5C). Also, H&E staining of major organs showed no obvious damage or inflammation in the Holo-Lf+4 Gy group compared with other groups (Figure S5D). Similarly, the GPX-4 expression level was not different between groups (Figure S5E). Thus, the anti-tumor efficacy of radiotherapy to the MDA-MB-231 tumor was augmented by Holo-Lf. Collectively, these data suggested that Holo-Lf sensitizes the MDA-MB-231 tumor to radiotherapy.

## Discussion

Our study discovered a paradoxical role of Holo-Lf and Apo-Lf in ferroptosis of MDA-MB-231 cells (Figure 9E). Holo-Lf induced ferroptosis, characterized by the accumulation of lethal ROS and lipid peroxidation products in MDA-MB-231 cells, whereas Apo-Lf had an opposite role in ROS generation and ferroptosis. Also, Holo-Lf had no obvious effect on the ferroptosis of MCF-7 cells. Besides its role in ferroptosis, Holo-Lf catalyzed the conversion of H_2_O_2_ and ameliorated the hypoxia microenvironment of MDA-MB-231 cells and tumors. Significantly, in combination with 4 Gy radiation, Holo-Lf induced more severe DNA damage to MDA-MB-231 cells and enhanced the efficacy of radiotherapy in MDA-MB-231 tumors.

As different iron-saturated forms of Lf, Apo-Lf and Holo-Lf exhibited distinct roles in many activities, including oxidative stress regulation. Apo-Lf is a potent iron chelator with the ability to bind labile iron and inhibit free radical formation, thus decreasing the intracellular oxidative stress. Shoji* et al.* reported that Apo-Lf could protect intestinal cells from H_2_O_2_-induced oxidative damage 53. Consistent with previous studies, we observed the antioxidant activity of Apo-Lf in the present study. Apo-Lf upregulated SLC7a11 expression and maintained GPX-4 activity without affecting its expression. However, this effect was weakened by increased iron saturation. The ferric iron in Holo-Lf could be released from Lf, especially in an acid tumor microenvironment 54. These loosely coordinated iron (labile iron) can catalyze ROS formation via Fenton chemistry and increase the intracellular oxidative stress. Therefore, Holo-Lf exhibited a pro-oxidant activity in MDA-MB-231 cells despite upregulating SLC7a11 expression.

Timmerman *et al.* investigated the sensitivity of different phenotypes of breast cancer cells to ferroptosis and found TNBC cells to be sensitive to glutamine restriction and ferroptosis 55. Iron is involved in many bioprocesses, such as cell respiration, oxygen metabolism, and energy metabolism. Thus, iron is an essential element for the replication, metabolism, and growth of normal cells, especially for cancer cells due to their high proliferation rate. Many cancer cells, including breast cancer cells, showed a trend towards increased iron absorption and decreased iron effluxion 56. However, excess iron resulted in increased oxidative stress and toxicity to cancer cells 57. Thus, maintaining iron homeostasis is crucial for cancer cells to avoid iron toxicity. Alonso *et al.* reported that MDA-MB-231 cells exhibited a lower total cytosolic iron concentration and a higher ferritin level than MCF-7 cells 58.

In the present study, Holo-Lf induced increased expression of TfR, ferritin, and ferroportin in MDA-MB-231 cells, indicating an increase in iron intake, storage, and export. By contrast, Holo-Lf increased ferritin expression but decreased that of TfR and ferroportin in MCF-7 cells. Consequently, Holo-Lf increased the total iron content more pronouncedly in MDA-MB-231 cells than MCF-7 cells. In line with this finding, Zalutski *et al.* observed that Lf induced more pronounced labile iron in ER-negative cell lines (MDA-MB-231 and MDA-MB-468 cells) than in ER-positive cell lines (MCF-7 and T47D cells) 59, indicating a lower iron processing capacity of MDA-MB-231 cells than MCF-7 cells. Besides iron homeostasis regulation, the redox balance capacity of cells was also different between these two breast cancer cells. Kwiatkowska *et al.* examined sensitivity of MDA-MB-231 and MCF-7 cells to the treatment of 3-bromopyruvate (3-BP), an inhibitor of glycolysis, and found that it significantly reduced the activity of GSH S-transferase and GSH reductase in MDA-MB-231 cells to a higher extent than MCF-7 cells 60. In our study, the GPX-4 protein and mRNA levels, as well as the GPX activity in MDA-MB-231 cells, were lower than in MCF-7 cells. The differences in iron homeostasis regulation and redox balance capacity between MDA-MB-231 and MCF-7 cells resulted in a higher ferroptosis sensitivity of MDA-MB-231 cells than MCF-7 cells; however, this result should be validated in other TNBC and non-TNBC cell lines.

Hypoxia, a pathophysiologic characteristic of solid tumors, attenuates radiotherapy's therapeutic efficacy by weakening the DNA damage of ionizing radiation and upregulating the HIF-1α expression. HIF-1α has been reported to activate the transcription of numerous hypoxia-inducible genes involved in tumor angiogenesis, proliferation, and glycolytic metabolism, thus enabling cancer cells to survive after radiotherapy 61-63. Therefore, the therapeutic efficiency of radiotherapy to cancers was remarkably abated by the tumor hypoxia microenvironment. Thus, reducing HIF-1α by improving the hypoxic microenvironment is expected to improve the tumor response to radiotherapy. A previous study discovered that Apo-Lf functioned as a hypoxia mimetic capable of stabilizing HIF-1α 64. Herein, we found that Holo-Lf ameliorated the hypoxia microenvironment and promoted the degradation of HIF-1α in hypoxic cells and tumors. More importantly, in cellular models, Holo-Lf induced increased ROS through Fenton reaction in the presence of H_2_O_2_. It is likely that the Holo-Lf effect on HIF-1α expression and ROS generation coordinately enhances the radiosensitivity of tumors in vivo. However, further investigations are required to study ROS generation in tumor tissues.

ROS is a common feature between ferroptosis and radiotherapy. It not only induces DNA damage, but also causes oxidative damage to the cell membrane and leads to ferroptosis of cancer cells 65. Lang *et al.* reported that radiotherapy promotes ferroptosis by repressing the expression of SLC7a11 66. Also, Lei *et al.* documented that radiotherapy induces ACSL4 expression, a lipid metabolism enzyme required for ferroptosis 67. Although the effect of Holo-Lf on SLC7a11 and ACSL4 expression after radiation was not examined in the present study, the cell membrane permeability of MDA-MB-231 cells was increased after treatment with Holo-Lf and 4 Gy radiation, a hallmark of lipid peroxidation and ferroptosis. Thus, the increased radiotherapy efficacy in MDA-MB-231 cells under high H_2_O_2_ or hypoxic environment was due to induction of ferroptosis and elevated radiosensitivity of cells by Holo-Lf. The effective *in vitro* concentration of Lf to initiate ferroptosis was about 400 µg/mL. For the *in vivo* study, we adopted a 2mg Lf dosage for each mouse to achieve the effective Lf concentration in the tumor site. However, the precise Lf dosage and its metabolism in the tumor site require further studies.

## Conclusion

The TNBC cell line MDA-MB-231 was more sensitive to ferroptosis than the non-TNBC cell line MCF-7. Holo-Lf released iron, increased ROS generation, and enhanced ferroptosis in MDA-MB-231 cells. However, Apo-Lf diminished ferroptosis in MDA-MB-231 cells by upregulating SLC7a11 expression. Furthermore, Holo-Lf catalyzed the decomposition of H_2_O_2_ to relieve the hypoxia microenvironment both in MDA-MB-231 cells and tumors and promoted ROS generation and DNA damage by radiotherapy. Finally, Holo-Lf enhanced the radiosensitivity of MDA-MB-231 cells and tumors.

## Supplementary Material

Supplementary figures and table.
