# Supplementary material for: Holo-lactoferrin: the link between ferroptosis and radiotherapy in triple-negative breast cancer
Source: Theranostics. 2021 Jan 1;11(7):3167–82. doi: 10.7150/thno.52028 (PMC7847686; doi:10.7150/thno.52028)
Supplement: Supplementary file 1 — Supplementary figures and table. [file thnov11p3167s1.pdf]

Table S1 Sequences of primers for qPCR.

Gene	Sequence
**SLC7a11**	Froward: 5’-TCTCCAAAGGAGGTTACCTGC-3’<br>Reverse: 5’ -AGACTCCCCTCAGTAAAGTGAC-3’
**GPX-4**	Froward: 5’ -TGTGGGCATCAATGGATTTGG-3’<br>Reverse: 5’ -ACACCATGTATTCCGGGTCAAT-3’
**Bax**	Froward: 5’ -CCCGAGAGGTCTTTTTCCGAG-3’<br>Reverse: 5’ -CCAGCCCATGATGGTTCTGAT-3’
**Bcl-2**	Froward: 5’ -GGTGGGGTCATGTGTGTGG-3’<br>Reverse: 5’ -CGGTTCAGGTACTCAGTCATCC-3’
**GAPDH**	Froward: 5’ -TGTGGGCATCAATGGATTTGG-3’<br>Reverse: 5’ -ACACCATGTATTCCGGGTCAAT-3’

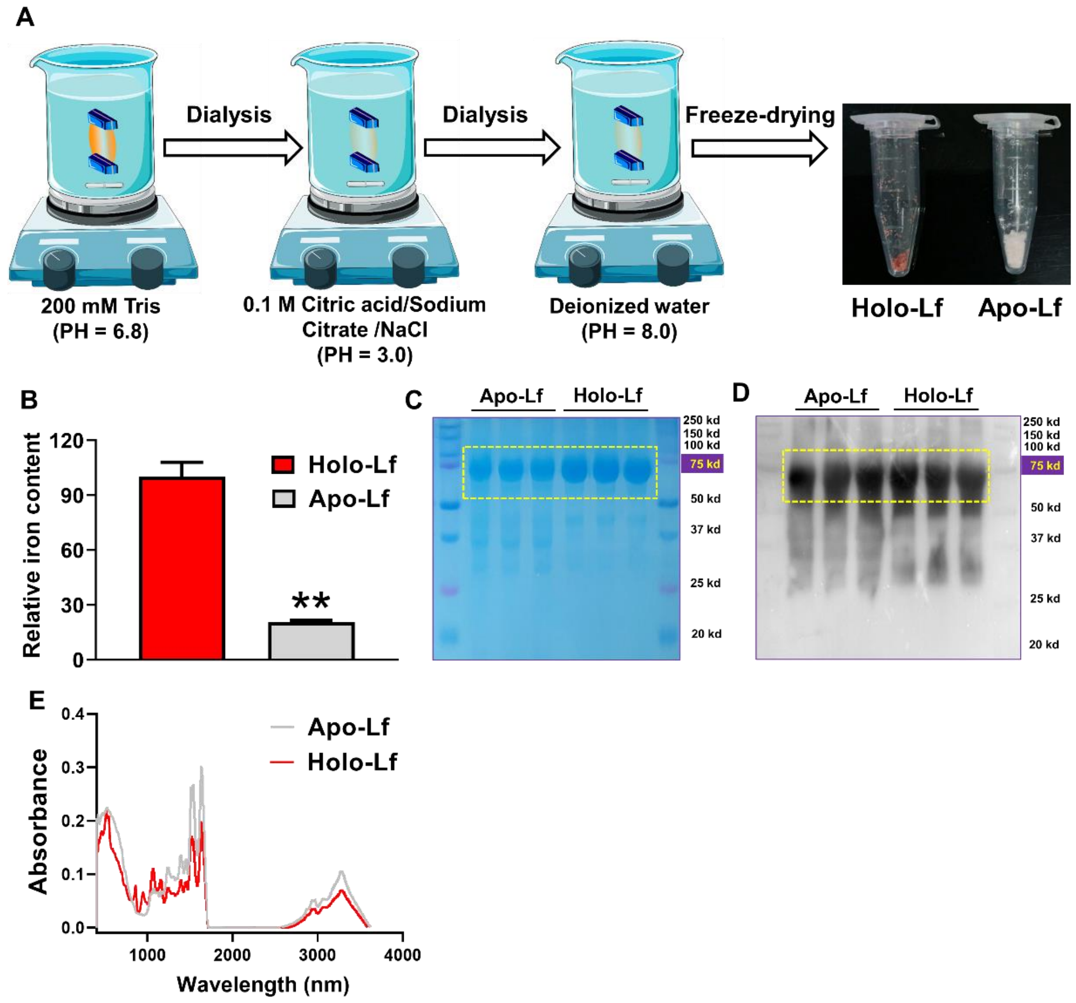

Figure S1 Preparation of Apo-Lf: (A) Diagrammatic sketch of preparation of Apo-Lf from Holo-Lf. (B) Relative iron content measured by ICP-MS (n = 3). (C) Coomassie blue staining of Apo-Lf and Holo-Lf (40 μg) after 12% SDS PAGE electrophoresis. (D) Western blotting image of Apo-Lf and Holo-Lf. Data were shown as mean±SD. (e) Fourier transformation infrared spectrometry (FTIR) of Apo-Lf and Holo-Lf. **Compared Holo-Lf group $P < 0.01$.

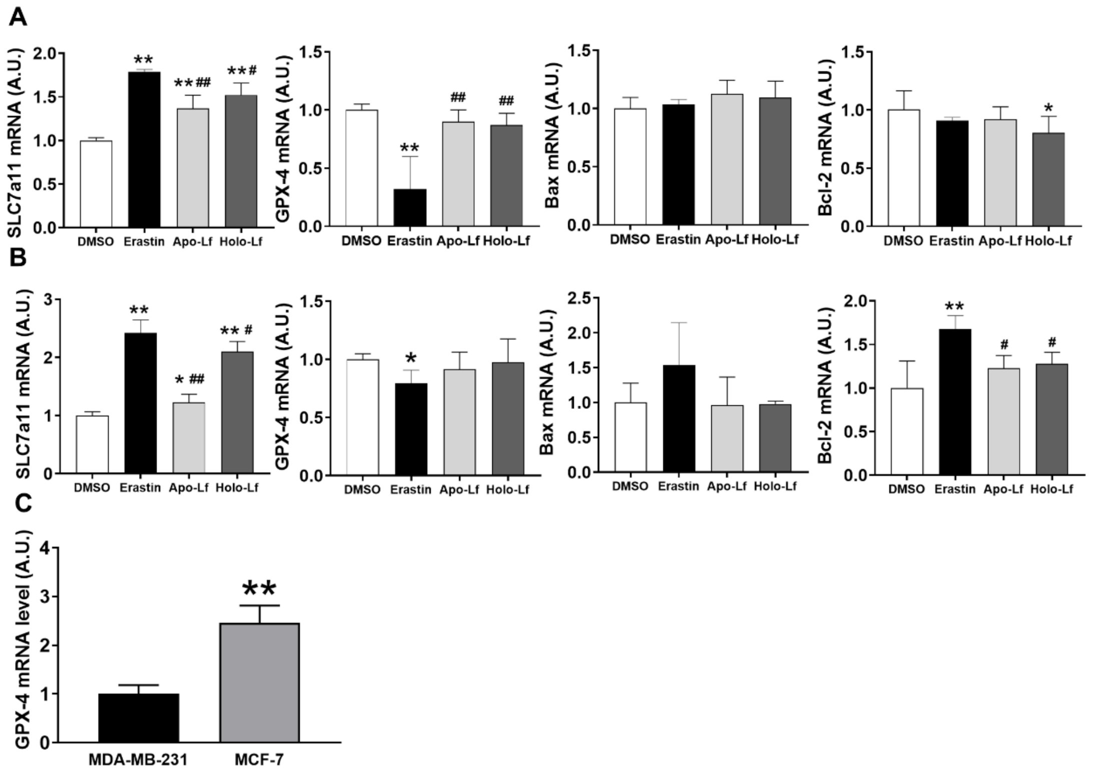

Figure S3 qPCR analysis of MDA-MB-231 and MCF-7 cells: (A) Relative gene expression level of SLC7a11, GPX-4, Bax and Bcl-2 in MDA-MB-231 after different treatments (n = 3). (B) Relative gene expression level of SLC7a11, GPX-4, Bax and Bcl-2 in MCF-7 after different treatments. Relative gene expression level was normalized to the expression level of appropriate gene in cells treated with DMSO (n = 3). (C) Relative gene expression level of GPX-4 between MDA-MB-231 and MCF-7 cells (n = 4). Relative gene expression level was normalized to the GPX-4 mRNA expression of MDA-MB-231 cells. Data were shown as mean±SD. *Compared with DMSO group $P < 0.05$. **Compared with DMSO group or MDA-MB-231 cells $P < 0.01$. #Compared with erastin group $P < 0.05$. ##Compared with erastin group $P < 0.01$.

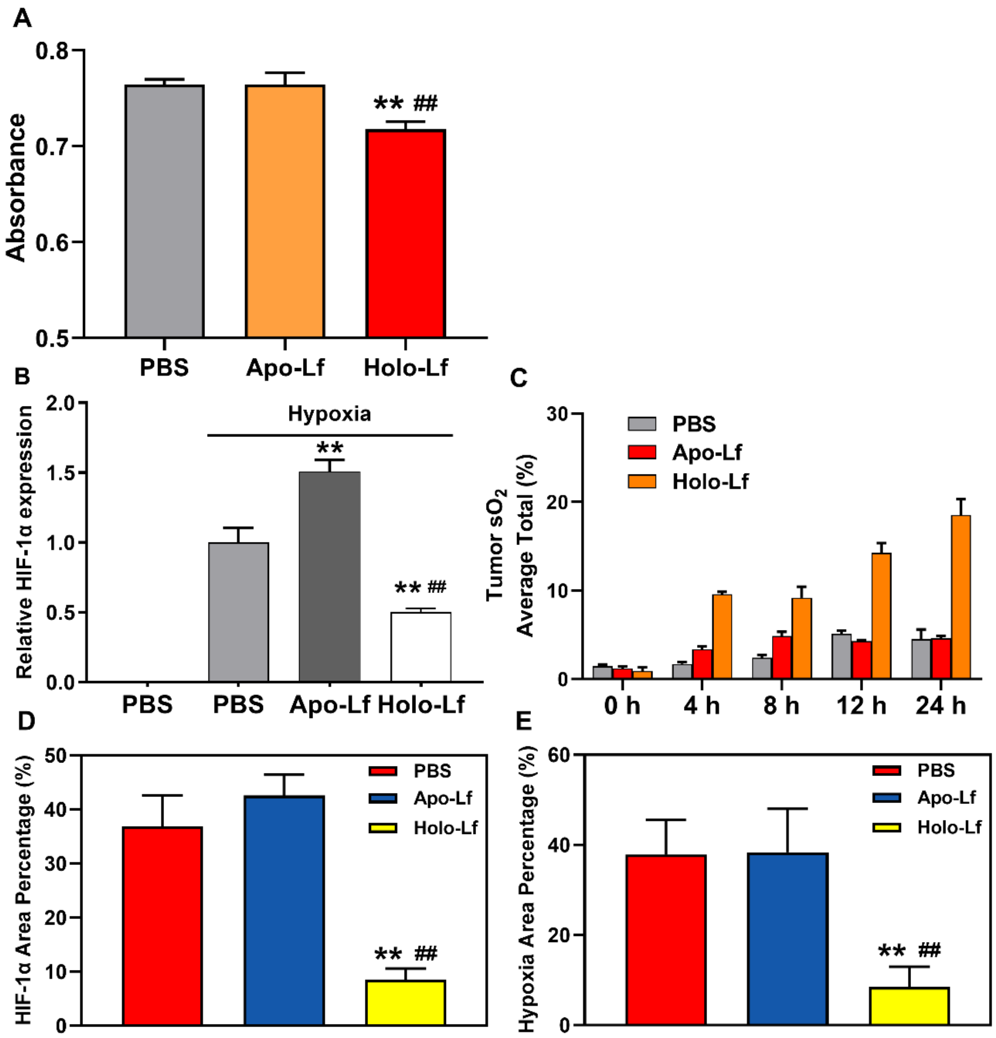

Figure S4 (A) The absorbance of H_2O_2 at 666 nm after incubated with PBS, Apo-Lf and Holo-Lf (n = 5). (B) Relative HIF-1α expression level of MDA-MB-231 cells after different treatments (n = 4). Total grey intensity of each protein was normalized to the grey intensity of appropriate protein in cells treated with PBS cultured in hypoxia condition. **Compared with hypoxia cells treated with PBS P<0.01. $^{\#\#}$Compared with hypoxia cells treated with Apo-Lf $P < 0.01$. (C) Tumor average total sO_2 of MDA-MB-231 tumor bearing mice injected with PBS, Apo-Lf and Holo-Lf at different time points (n = 5). (D) Percentage of HIF-1α positive areas (n = 4). (E) Percentages of hypoxia positive areas (n = 4). **Compared with PBS group $P < 0.01$. $^{\#\#}$Compared with Apo-Lf group $P < 0.01$.

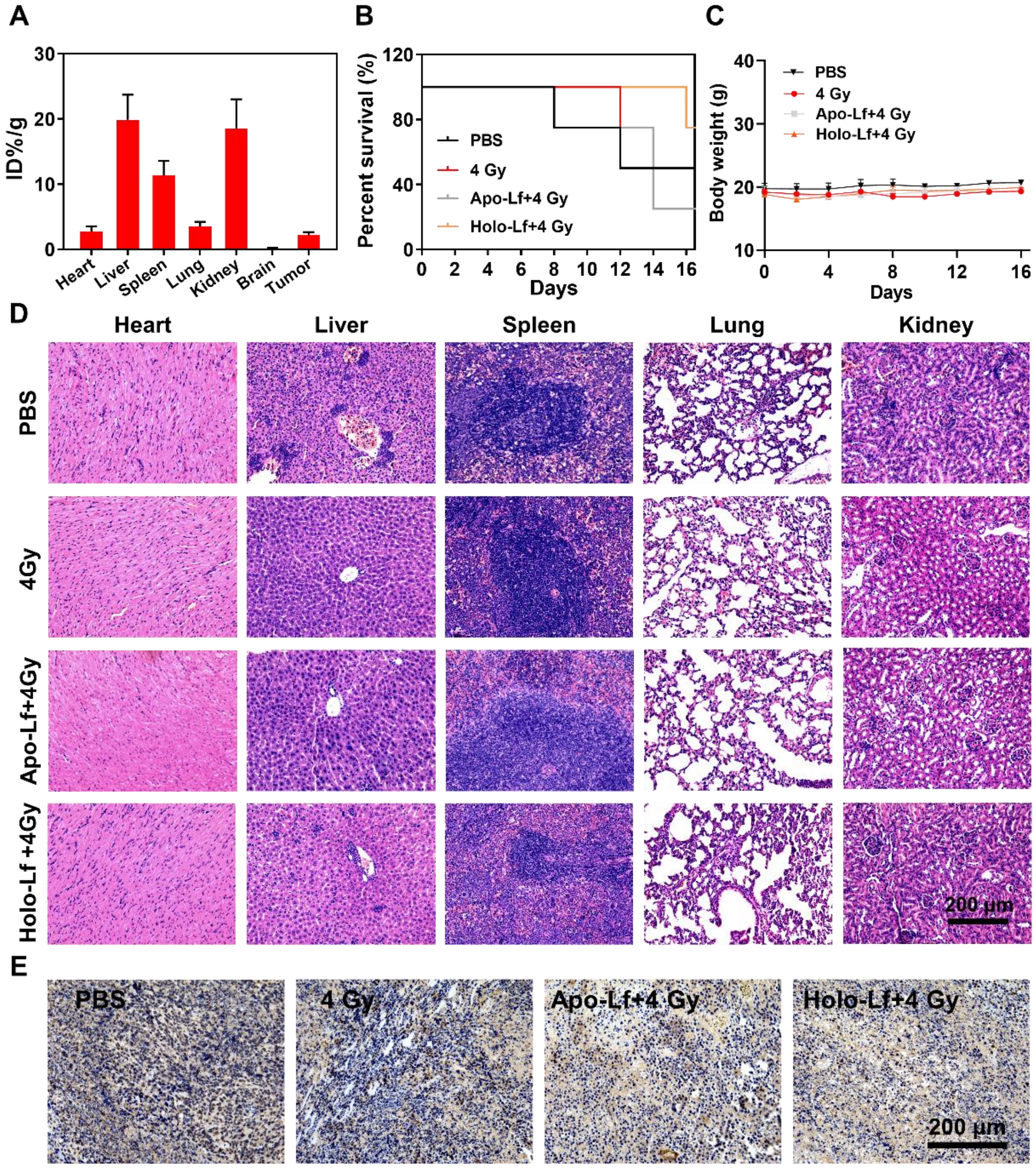

Figure S5 (A) Quantitative analysis of major organs radiation activities 24 h after i.v. injected with ^{125}I labeled Holo-Lf (n = 3). (B) Body weight changing curves of mice treated with PBS, 4 Gy, Apo-Lf+4 Gy and Holo-Lf+4 Gy (n = 4). (C) Survival rates of mice treated with PBS, 4 Gy, Apo-Lf+4 Gy and Holo-Lf+4 Gy (n = 4). (D) H&E stained images of major organs obtained from different treatment groups: heart, liver, spleen, lung, and kidney. (E) GPX-4 expression was detected by IHC in mouse tumor tissues of each group. Data were shown as mean±SD.
